# Distinct aberrations in cerebral pain processing differentiating patients with fibromyalgia from patients with rheumatoid arthritis

**DOI:** 10.1097/j.pain.0000000000002387

**Published:** 2021-06-28

**Authors:** Angelica Sandström, Isabel Ellerbrock, Monika Löfgren, Reem Altawil, Indre Bileviciute-Ljungar, Jon Lampa, Eva Kosek

**Affiliations:** a Department of Clinical Neuroscience, Karolinska Institutet, Stockholm, Sweden; b Department of Neuroradiology, Karolinska University Hospital, Stockholm, Sweden; c Department of Clinical Sciences, Karolinska Institutet, Danderyd University Hospital, Stockholm, Sweden; d Department of Medicine, Rheumatology Unit, Center for Molecular Medicine (CMM), Karolinska Institutet, Karolinska University Hospital, Stockholm, Sweden; e Department of Surgical Sciences/Pain Research, Uppsala University, Uppsala, Sweden

**Keywords:** Functional magnetic resonance imaging (fMRI), Rheumatoid arthritis, Fibromyalgia, Cerebral pain processing, Functional connectivity, Inferior parietal lobe, Frontoparietal network, Sensorimotor network

## Abstract

The current study used functional magnetic resonance imaging to directly compare disease-relevant cerebral pain processing in well-characterized patient cohorts of fibromyalgia (FM, nociplastic pain) and rheumatoid arthritis (RA, nociceptive pain). Secondary aims were to identify pain-related cerebral alterations related to the severity of clinical symptoms such as pain intensity, depression, and anxiety. Twenty-six patients with FM (without RA-comorbidity) and 31 patients with RA (without FM-comorbidity) underwent functional magnetic resonance imaging while stimulated with subjectively calibrated painful pressures corresponding to a pain sensation of 50 mm on a 100-mm visual analogue scale. Stimulation sites were at the most inflamed proximal interphalangeal joint in the left hand in patients with RA and the left thumbnail in patients with FM, 2 sites that have previously been shown to yield the same brain activation in healthy controls. The current results revealed disease-distinct differences during pain modulation in RA and FM. Specifically, in response to painful stimulation, patients with FM compared to patients with RA exhibited increased brain activation in bilateral inferior parietal lobe (IPL), left inferior frontal gyrus (IFG)/ventrolateral prefrontal cortex (vlPFC) encapsulating left dorsolateral prefrontal cortex, and right IFG/vlPFC. However, patients with RA compared to patients with FM exhibited increased functional connectivity (during painful stimulation) between right and left IPL and sensorimotor network and between left IPL and frontoparietal network. Within the FM group only, anxiety scores positively correlated with pain-related brain activation in left dorsolateral prefrontal cortex and right IFG/vlPFC, which further highlights the complex interaction between affective (ie, anxiety scores) and sensory (ie, cerebral pain processing) dimensions in this patient group.

## Abstract 2

Supplemental Digital Content is Available in the Text.

Patients with rheumatoid arthritis > patients with fibromyalgia exhibited reduced pain-related brain activation in parietal and frontal regions. However, FM > RA exhibited disrupted pain-related connectivity between parietal and somatosensory regions.

## 1. Introduction

Rheumatoid arthritis (RA) is a nociceptive pain disorder characterized by peripheral joint inflammation that causes pain, swelling, stiffness, and destruction of the affected joints.^37^ Despite excellent control of inflammation in RA joints, many patients with RA continue to report pain and there is a high prevalence of concomitant fibromyalgia (FM) in RA compared with the general population.^6,8,26,30,31^ Fibromyalgia is a nociplastic pain disorder^24^ characterized by altered pain processing, with widespread pain, generalized hypersensitivity to sensory stimuli, often in combination with fatigue, disturbed sleep, memory difficulties, and psychological distress.^48^ It has been suggested that, in certain patients with RA, peripheral inflammatory processes sensitise the central nervous system (CNS) via pronociceptive pathways^39^ and drives the CNS towards a nociplastic state,^18^ a state that characterizes FM.^24^ Yet, contemporary research indicates fundamental differences in central pain processing in these 2 patient populations when they are well characterized and compared with healthy controls (HCs).

Well-characterized RA patients (without concomitant FM) show normal function of descending pain inhibition, such as conditioned pain modulation^33^ and exercise-induced hypoalgesia.^13,34,38^ These observations relate to normal cerebral pain processing during painful stimulation at nonaffected sites (ie, thumbnail) in patients with RA compared to HC.^43^ However, painful stimulation at a disease-affected inflamed finger joint yielded decreased brain activation in primary and secondary somatosensory cortex and insula in RA compared with HCs.^43^ High levels of inflammation in RA has been associated with reduced gray matter of the inferior parietal lobe (IPL), as well as increased functional connectivity between the left IPL and multiple brain networks such as the frontoparietal network (FPN), salience network, and the default mode network.^46^

In well-characterized FM patients (without concomitant RA), abnormalities in the function of endogenous descending pain inhibition has repeatedly been demonstrated, including conditioned pain modulation^25,41^ and exercise induced hypoalgesia.^25,27,40^ Neuroimaging studies have linked dysfunctional pain inhibition in FM to CNS aberrations by, for example, demonstrating imbalances in levels of neurotransmitters that affect pain and sensory transmission^45,48^ and reduced brain activation and connectivity between regions related to descending pain modulatory brain regions (eg, anterior cingulate cortex and periaqueductal gray) in patients with FM compared to HC during painful stimulation.^21,22^ Functional connectivity analyses of the FM brain at rest have revealed a reduced coupling between pain and somatosensory-related brain regions (eg, thalamus and insula to primary sensory and motor cortices) compared with HCs,^12^ as well as an overall more variable/less stable global network architecture in patients with FM compared to HCs, which was more pronounced among patients with higher clinical pain.^28^

The main aim of the current exploratory study was to use functional magnetic resonance imaging (fMRI) to directly compare brain function and functional connectivity during painful pressure stimulation over disease-relevant areas in well-characterized cohorts of patients with RA and FM to identify disease-specific aberrations in cerebral pain processing. Secondary aims were to identify cerebral pain modulatory alterations related to the severity of clinical symptoms such as pain intensity, depression, and anxiety in these patient groups.

## 2. Methods

### 2.1. Participants

#### 2.1.1. Rheumatoid arthritis cohort

Patients with RA were initially recruited through the rheumatology clinic at the Karolinska Hospital in Stockholm, Sweden, to participate in a randomized, placebo-controlled trial investigating the effects of a tumour necrosis factor (TNF-alpha) inhibitor on inflammation and pain (the PARADE study; www.clinicaltrials.gov; [identifier NCT01197144, EudraCT 2009-017163-42]). In this article, we only include baseline data (ie, before initiation of drug or placebo). Baseline data from the RA cohort have been published previously.^11,43^ For full RA baseline study protocol and flowchart of the recruitment process, see Ref. 43. In total, 34 patients with RA completed baseline fMRI. Three patients with RA were excluded due to technical issues such as technical failure or excessive head motion. The final sample included in the current analyses consisted of 31 patients with RA (6 male).

Patients with RA were screened by a MD to ensure that included patients fulfilled the ACR 1987 classification criteria for RA (Arnett et al., 1988), ≥18 years, had clinical indication for use of TNF-blockers, and were willing to participate and approved by MD for MR examination. Exclusion criteria were FM comorbidity, left-handedness, neurological disease, severe cardiovascular disease, latent tuberculosis, comorbid depression, ongoing treatment with antidepressants, claustrophobia, pregnancy, previous treatment with biologics, or other motives based on the assessment of the responsible physician. The most inflamed finger joint was assessed in each individual patient by a specialist in rheumatology. The study conformed with Swedish legislation regarding clinical pharmacological trials, and necessary permit from the Swedish medical products agency was obtained. The regional ethics committee in Stockholm approved the study, and verbal as well as written informed consent was obtained from all participants.

#### 2.1.2. Fibromyalgia cohort

Patients with FM were initially recruited to participate in a multicentre longitudinal intervention study investigating the cerebral effects of 15-week physical exercise or relaxation therapy (ClinicalTrials.gov identification number: NCT01226784).^29^ Imaging data regarding resting state and cerebral activation during a cognitive task from the FM cohort have previously been published elsewhere.^11,12,35,36^ In this article, we only include previously unpublished pain-related baseline data (ie, before initiation of intervention) from the Stockholm cohort, ie, the only centre that had collected fMRI data. In total, 26 female patients with FM were eligible for fMRI and included in the current fMRI analysis, none excluded.

Patients with FM were physically examined by a specialist in rehabilitation medicine to ensure that they fulfilled the inclusion criteria, ie, meeting the ACR-1990 classification criteria for FM (Wolfe et al., 1990), female sex, and being of working age (20-65 years). Exclusion criteria were other primary causes of pain than FM, comorbid severe somatic disorder (including RA or osteoarthritis in hip or knee), psychiatric disorders, high blood pressure (>160/90 mm Hg), high consumption of alcohol (Audit >6), participation in any other rehabilitation program within the past year, contemporary regular resistance exercise training or relaxation exercise training ≥2/week, inability to understand or speak Swedish, and not being able to refrain from NSAID, analgesics, or hypnotics for 48 hours before examinations. The study was conducted in accordance with the Declaration of Helsinki, with approval from the regional ethics committee in Stockholm (2010/1121-31/3). All participants were given written and oral information, and written consent was obtained.

#### 2.1.3. Healthy controls

The HCs were recruited in parallel to the patients through noticeboard advertisements primarily at the hospital campus with an attempt to sex- and age-match HCs to the patients with RA. Study personnel, behavioral testing, scan protocol, and facilities used to examine HCs were the same as for patients with RA and FM. Exclusion criteria for the HCs were identical to those of the patients with RA, with the additional exclusion criteria of recurrent pain problems (including RA and FM) and regular use of over-the-counter pain medication or prescribed analgesics. This particular HC group has been included in previous studies.^11,43^ In total, 26 HCs fulfilled the above-mentioned criteria. However, 1 HC was excluded due to technical issues and 2 HCs exhibited excessive head motion within the fMRI scanner, leaving 23 HC for complete data analysis. For more information on the method for determining excessive head motion, see section 3.1.

### 2.2. Questionnaires

Additional measurements collected included disease duration (in months), spontaneously ongoing pain rated through visual analogue scale (VAS), hospital anxiety and depression scale (HADS), fibromyalgia impact questionnaire (FIQ, FM only), and health assessment questionnaire (HAQ, RA only).

Visual analogue scale pain was used to assess self-rated clinical, spontaneous ongoing pain throughout the body, through a one-item question. All patients (RA and FM) were asked to rate their current pain through putting a mark on a 0 to 100 mm VAS scale (ranging from 0 “no pain” to 100 “worst imaginable pain”).

Hospital anxiety and depression scale is a 14-item self-assessment divided into 2 subscales: anxiety (HADS-a) and depression (HADS-d) consisting of 7 items each.^53^ Subjects rate on a 4-point Likert scale (ranging from 0 to 3). A total score of less than 7 points of either subscale is of no clinical relevance, 8 to 10 points indicate intermediate levels, and over 11 points indicate clinical relevant levels of depression or anxiety.

Fibromyalgia impact questionnaire is an instrument developed for clinical and research settings to assess the current health status in individuals with FM.^19^ The 20-item instrument measures pain, stiffness, fatigue, morning tiredness, work status (missed days of work and difficulty), depression, anxiety, and well-being over the past week.

Health assessment questionnaire is an instrument developed to assess the level of difficulty that patients with RA have experienced in the past week when dressing and grooming, arising, eating, walking, reaching, gripping, and during hygiene routines and common daily activities. The instrument contains 8 sections with 2 or 3 items in each section. Scoring within each section ranges from 0 to 3 (0 = without any difficulty; 1 = with some difficulty; 2 = with much difficulty; 3 = unable to do).^42^

### 2.3. Procedure day 1: subjective calibration of pressure pain stimuli

After inclusion, patients with FM, patients with RA, and HCs visited the same facility (Karolinska Hospital, Stockholm, Sweden) on 2 consecutive days within the same data-collection period. All participants (RA, FM, and HCs) were individually assessed for subjective pressure pain sensitivity during their first visit using an automated, pneumatic, computer-controlled stimulator that applies pressure via a plastic piston with a 1-cm^2^ hard rubber probe that was placed at the left thumbnail in FM and RA, as well as at the clinically most affected left hand proximal interphalangeal (PIP) joint of patients with RA only (PIP2 n = 25; PIP3 n = 6). For full calibration protocol, refer the study by Sandström et al.^43^ In brief, all participants were individually calibrated for subjective pain ratings to ultimately determine which individual pressure (kPa) corresponded to a subjective rating of 50-mm VAS. First, the participants received one ascending series of pressure stimuli (with increasing steps of 50 kPa) to determine the pressure pain threshold (PPT, first VAS > 0 mm) and stimulation maximum (SM, first VAS > 60 mm). Next, participants received 3 randomized series, in which 5 different pressure intensities were calculated and delivered within the range of each patient's pressure pain threshold and stimulation maximum pressures. After each pressure, the participants were instructed to rate the pain intensity on a VAS (ranging from 0 = no pain, to 100 = worst imaginable pain). All pressures were applied for 2.5 seconds with 30-second intervals. Finally, a polynomial regression function was applied to fit the data, using the 15 ratings from the randomized series of pressure pain, to determine each participant's representation of 50-mm VAS (designated as P50) (for further information, see Refs. 21 and 43).

### 2.4. Procedure day 2: functional magnetic resonance imaging acquisition

On the second day, participants were placed inside the scanner. First, anatomical MR scans were collected. Then, participants underwent the same computerized stimulation paradigm that triggered the pressure probe stimulation apparatus while functional MR images were collected. All participants were instructed to focus on the pressures and not to use any coping or distraction techniques. Patients with FM were stimulated on their left thumbnail with pressures corresponding to their previously individually calibrated painful pressure (P50) and a nonpainful pressure stimulus (50 kPa) during 2 runs. The painful and nonpainful pressures were delivered in a pseudorandomized order within each of these 2 runs. Healthy controls and patients with RA were stimulated with their subjectively calibrated P50 and a nonpainful pressure (50 kPa) on their left thumbnail during 2 runs, and stimulated on their left joint during 2 runs. The painful and nonpainful pressures were delivered in a pseudorandomized order within each of these runs. The pressure stimulator probe only stimulated one site (joint or thumb) at a time. Hence, stimulation runs in patients with RA and HCs alternated between joint and thumb in a counterbalanced order (eg, joint, thumb, joint, and thumb).

Each run consisted of 30 pressure stimuli (15 painful and 15 nonpainful stimuli) that were delivered for 2.5 seconds in a pseudorandomized order. Pressures were jittered over time with a mean interval between onsets of stimuli of 15 seconds (range 10-20 seconds). Total duration of each run was 8.15 minutes. At the end of the MR session, resting-state data were collected in both patient cohorts (published elsewhere^11,12^). MR images were acquired with a 3T General Electric 750 MR scanner installed at the MR Research Center, Karolinska Institutet, Stockholm, using a 32-channel head coil. Functional images covering the whole brain were acquired using a T2*-weighted single-shot gradient echo planar imaging sequence. The following parameters were used in both cohorts: flip angle = 90°, 96 × 96 matrix size, FOV = 288 × 288 mm, TR/TE =3000/30 ms, interleaved axial slice acquisition, 56 slices, and 3-mm slice thickness. Anatomical MR scans were acquired in both cohorts with a high-resolution BRAVO 3D T1-weighted image sequence (1 × 1 × 1-mm^3^ voxel size). Anatomical (T2-weighted) scans were investigated by neuroradiologist for clinical abnormalities.

## 3. Statistics

Behavioural data on pressure pain sensitivity were summarized and analysed in R (version 3.5.1) using two-sample *t* test.

### 3.1. Functional imaging data analysis

Imaging data analyses were performed using the Statistical Parametric Mapping 12 software^15^(http://www.fil.ion.ucl.ac.uk/spm/software/spm12/) running under Matlab (The MathWorks, Natick, MA, version R2019a). Before preprocessing, scans were manually reoriented to the anterior/posterior commissure line to improve the coregistration and normalization process. Functional images were spatially realigned using a six-parameter affine transformation and registered to the mean. Individual structural images were coregistered with functional images. Coregistered images were normalized to Montreal Neurological Institute (MNI) space and spatially smoothed using an 8-mm full-width-half-maximum Gaussian kernel. Frame-wise displacement was used to assess head motion from one volume to the next, converting rotational displacements (sum of the absolute values of the derivatives of the 6 realignment parameters) from degrees to millimetres by calculating displacement on a sphere with a 50-mm radius. Excessive head motion was considered for images exceeding frame-wise displacement >0.5, in >15% of the images in the baseline (preintervention) scanning sessions in RA and FM. Likewise, excessive head motion was considered for HCs' images exceeding frame-wise displacement >0.5, in >15% of the images in their single scanning session (as no intervention was applied in HCs).

First-level General Linear Model included regressors of interest convolved with the canonical haemodynamic response function separate for stimuli (ie, pain and sensory) and body site (disease-relevant thumbnail in FM; and disease-relevant joint as well as disease irrelevant thumbnail in RA). Motion parameters were added as regressors of no interest. To focus our analyses on cerebral pain processing in response to clinically relevant painful and sensory stimulation in these 2 chronic pain cohorts, we compared left hand thumbnail site stimulation sessions in FM with left hand joint site simulation sessions in RA. This decision was based on previous studies, in which we have demonstrated (using the same RA cohort) increased pain sensitivity and altered cerebral pain-related processing during painful stimulation at the disease-relevant most inflamed finger joint in RA vs HCs. However, pain sensitivity and cerebral pain processing remained normal when painfully stimulated at the control site (ie, unaffected disease irrelevant thumbnail) in RA vs HCs.^43^ In FM, significantly lower pressure pain thresholds and altered cerebral processing have been detected after thumbnail stimulation compared with HCs,^17,21^ suggesting that the thumbnail may serve as a clinically relevant stimulation site in patients with FM. Potential contributions of age, sex, and stimulus intensity (kPa) were investigated through regression analyses. Age, but not sex and stimulus strength, significantly influenced pain-related brain imaging data. Therefore, we included age as a regressor of no interest throughout all second-level analyses (univariate and psychophysiological interaction).

Second-level two-sample *t*-tests were used to investigate between-group differences in cerebral processing of contrasts [Pain], [Sensory], and [Pain-Sensory] pressure stimuli. One-sample *t*-tests were used to investigate within-group cerebral processing of the same contrasts. Furthermore, age was used as a covariate of no interest throughout all whole-brain correlational analyses and performed on contrast [Pain-Sensory] with the following parameters: disease duration (in months), VAS, P50 kPa, HADS-anxiety, HADS-depression, FIQ (FM only), and HAQ (RA only). Correlational analyses were performed within groups as well as between groups for the parameters that were shared across groups.

Statistical significance was considered for clusters surviving cluster-level family-wise error correction for multiple comparisons (*P* < 0.05) over the entire brain at an initial statistical threshold of *P* < 0.001, uncorrected with at least 20 contiguously activated voxels. No regions-of-interest analyses were used. Cluster brain activation was localized through using MNI stereotactic atlas coordinates [x, y, z] and labelled through the Automated Anatomical Labeling digital atlas in MRIcron.

#### 3.1.1. Psychophysiological interaction analysis

To further investigate between-group differences in cerebral processing of painful stimuli, a secondary PPI analysis^14^ was performed based on the univariate fMRI results of FM>RA [Pain-Sensory]. Psychophysiological interaction can be considered as a simple connectivity model that investigates the interaction between an experimental condition (psychological parameter: in this context, applied pressure stimuli) and a predetermined source region or a volume of interest (physiological parameter: ie, the BOLD fMRI signal time series).^14^ We chose to establish secondary task-based functional connectivity from our main between-group findings (ie, FM>RA [pain-sensory]), also supported by previous literature suggesting a specific role of the right and left IPL in rheumatic conditions.^2,23^ Specifically, an 8-mm diameter spherical volume of interest was defined in the left IPL [MNI −62, −38, 38] and right IPL [MNI 56, −24, 24].

## 4. Results

### 4.1. Behavioural results

**Figure 1.**
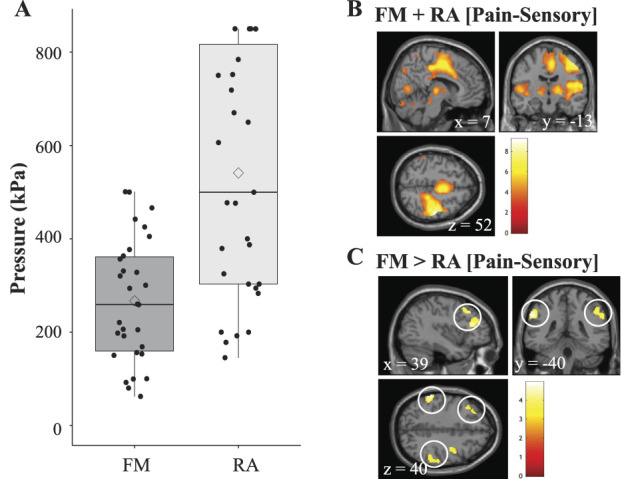
(A) Boxplots illustrate average pressure (kPa) corresponding to subjective pain ratings of 50 mm on a visual analogue scale (VAS) when stimulated with painful pressure. Patients with rheumatoid arthritis (RA) were stimulated at the most inflamed proximal phalangeal joint, left hand, and patients with fibromyalgia (FM) at the left thumbnail (We have previously demonstrated that patients with RA had increased pain sensitivity at the inflamed joint, but normal pain sensitivity at the nonaffected thumbnail compared to HC^43^). Horizontal lines within boxes represent median values. Diamond-shaped dots represent mean values. Box top and bottom frames represent 25th and 75th percentiles, respectively. Whiskers represent minimum and maximum values. Black dots represent individual painful pressure values. (B) All patients' (collapsed groups) brain activation in response to painful minus sensory (50 kPa) pressure stimulation. Significantly activated clusters across all patients encapsulated several pain-related brain regions such as right S1, right M1, bilateral S2, MCC, bilateral thalamus, and insula. (C) Brain regions where FM compared to RA exhibited increased brain activation in response to painful minus sensory stimulation. Significantly activated clusters were located in bilateral IPL, left dlPFC, and right IFG. The depicted brain activation was derived from a whole-brain statistical map corrected for multiple comparisons using FWE correction *P*_*FWE*_ <0.05 at an initial threshold of *P* < 0.001 uncorrected with 20 contiguously activated voxels. dlPFC, dorsolateral prefrontal cortex; IFG, inferior frontal gyrus; IPL, inferior parietal lobe; FWE, family-wise error; HC, healthy control; kPa, kilopascal; M1, primary motor cortex; MCC, midcingulate cortex; S1, primary somatosensory cortex; S2, secondary somatosensory cortex.

A total of 26 patients with FM and 34 patients with RA completed fMRI. Three patients with RA were excluded due to technical difficulties such as showing excessive head motion during scan. None of the patients with FM were excluded. Therefore, the final sample included for all subsequent analyses consisted of 26 FM (mean age ± SD = 49 ± 8; 26 females) and 31 RA (mean age ± SD = 54 ± 14; 25 females). Demographic and behavioural patient data can be found in Table 1. There were no significant group differences between patients with FM and patients with RA regarding age, body mass index, or clinical pain (VAS). The pressure corresponding to P50 was significantly lower in patients with FM, compared to patients with RA (Table 1 and Fig. 1A), and patients with FM had significantly longer disease duration and higher ratings of depression and anxiety (Table 1). Information regarding HC painful pressure sensitivity at the (left hand) can be found in supplementary table 1 (available at http://links.lww.com/PAIN/B421).

**Table 1:** Patient characteristics.

Patient characteristics	FM, n = 26	RA, n = 31	Group comparison
Age, years mean (SD, min-max)	49 (8, 25-64)	54 (14, 23-72)	*P* = 0.1
Gender (*n,* F/M)	26/0	25/6	—
Disease duration months mean (SD, min-max)	99 (46, 12-192)	43 (38, 3-120)	*P* < 0.001
BMI mean (SD, min-max)	26 (5, 19-36)	25.54 (5, 17-39)	*P* = 0.4
P50 kPa mean (SD, min-max)	271 (137, 62-501)	541 (257, 145-850)	*P* < 0.001
VAS current mean (SD, min-max)	47 (22, 8-92)	37 (26, 0-83)	*P* = 0.1
FIQ mean (SD, min-max)	65 (13, 42-95)	—	—
Tender points mean (SD, min-max)	16 (2, 12-18)	—	—
HAQ mean (SD, min-max)	—	0.67 (0.58, 0-2.13)	—
DAS-28 ESR	—	4.96 (1.19, 2.71-7.78)	—
HADS-anxiety mean (SD, min-max)	9 (4, 0-18)	5 (4, 0-16)	*P* < 0.001
HADS-depression mean (SD, min-max)	8 (4, 3-16)	4 (3, 0-10)	*P* < 0.001

BMI, body mass index; DAS, disease activity score; ESR, erythrocyte sedimentation rate; FIQ, fibromyalgia impact questionnaire; FM, fibromyalgia; HADS, hospital anxiety and depression scale; HAQ, health assessment questionnaire; F, female; kPa, kilopascal; M, male; P50 kPa, painful pressure corresponding to VAS 50; RA, rheumatoid arthritis; VAS, visual analogue scale (mm).

### 4.2. Neuroimaging results

To ensure proper cerebral pain-related activation, contrast [Pain-Sensory] was investigated across all subjects. The contrast yielded increased pain-related brain activation in clusters encapsulating right postcentral gyrus (primary sensory cortex, S1), rolandic operculum (secondary somatosensory cortex, S2), precentral gyrus (primary motor cortex, M1), midcingulate cortex (MCC), thalamus, insula, cerebellum, and cuneus (Fig. 1B). Whole-brain between-group comparison univariate analyses revealed significantly more brain activation in FM>RA [Pain-Sensory] in right and left IPL, left inferior frontal gyrus (IFG) pars triangularis, extending to dorsolateral prefrontal cortex (dlPFC) and right IFG pars opercularis (Fig. 1C and Table 2). There were no brain regions where RA>FM [Pain-Sensory] exhibited increased activation in the univariate whole-brain analyses.

**Table 2:** Univariate results of contrast [pain-sensory].

BOLD [pain-sensory]	x	y	z	k/E	*z*-score	few
FM>RA [pain-sensory]						
L supramarginal gyrus/inferior parietal lobe	−62	−38	38	367	4.47	0.030
L IFG pars triangularis, *encapsulating L midfrontal gyrus (dlPFC)*	−38	32	20	378	4.25	0.027
R IFG pars opercularis	44	10	22	528	4.13	0.008
R supramarginal gyrus/inferior parietal lobe	56	−24	24	339	3.76	0.038
RA > FM [pain-sensory]						
n/s						
FM [pain-sensory]						
R supramarginal gyrus, *encapsulating R insula, IFG pars opercularis, rolandic operculum (S2), superior temporal pole, precentral gyrus (M1)*	58	−24	22	11774	5.18	<0.001
L cerebellum crus 2, *extending to L cerebellum 8*	−4	−84	−26	3231	4.92	<0.001
L insula	−46	8	−8	1452	4.81	<0.001
L supramarginal gyrus	−62	−36	26	1603	4.62	<0.001
R cerebellum crus 1	44	−64	−32	865	4.34	<0.001
L midfrontal gyrus (dlPFC)	−36	44	26	307	3.73	0.049
RA [pain-sensory]						
R postcentral gyrus (S1)	50	−20	52	2446	6.14	<0.001
L rolandic operculum	−46	−22	16	2440	6.13	<0.001
L cerebellum 4 5	−16	−54	−20	789	5.65	<0.001
R rolandic operculum (S2)	42	−18	16	1858	5.31	<0.001
R midcingulate cortex	6	−10	50	1187	5.28	<0.001
R thalamus	12	−18	−4	290	5.21	0.042

dlPFC, dorsolateral prefrontal cortex; FM, fibromyalgia; FWE, family-wise error; IFG, inferior frontal gyrus.

**Figure 2.**
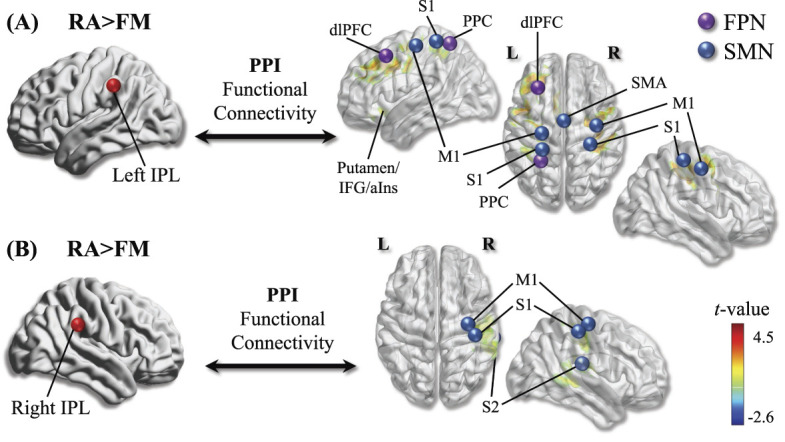
Differences in task-based connectivity between patients with rheumatoid arthritis (RA) and FM during [Pain-Sensory] stimulation. Specifically, (A) patients with RA compared to FM exhibited increased task-based connectivity between left inferior parietal lobe (IPL) (red dot) and brain regions involved in FPN (purple dots) and SMN (blue dots). (B) Patients with RA compared to FM exhibited increased task-based connectivity between right IPL (red dot) and SMN (blue dots). The depicted brain activation (including PPI) was derived from a whole-brain statistical map corrected for multiple comparisons using FWE correction *P*_*FWE*_ <0.05 at an initial threshold of *P* < 0.001 uncorrected with 20 contiguously activated voxels. dlPFC, dorsolateral prefrontal cortex; FM, fibromyalgia (patients); FWE, family-wise error; IPL, inferior parietal lobe; IFG, inferior frontal gyrus; aIns, anterior insula; M1, primary motor cortex; PPC, posterior parietal cortex; PPI, psychophysiological interaction; RA, rheumatoid arthritis (patients); S1, primary somatosensory cortex; S2, secondary somatosensory cortex; SMN, sensorimotor network.

Psychophysiological interaction seeding from the left IPL revealed increased functional connectivity for RA>FM [Pain-Sensory] between left IPL and M1 encapsulating bilateral S1, left dlPFC, and supplementary motor area; left S1; and left putamen encapsulating anterior insula (Fig. 2A and Table 3). Psychophysiological interaction seeding from the right IPL revealed increased functional connectivity for RA > FM [Pain-Sensory] with S1 and S2 (Fig. 2B and Table 3). No increment in functional connectivity was found for FM > RA [Pain-Sensory] when seeding from either left or right IPL. Within-group [Pain-Sensory] fMRI results can be found in Table 2. For more information regarding between- and within-group fMRI results investigating [Pain Only] and [Sensory Only], see supplementary tables 2 and 3, respectively (available at http://links.lww.com/PAIN/B421). For information regarding HCs, see supplementary table 1 (available at http://links.lww.com/PAIN/B421), which includes information regarding kPa pressures in HC, as well as brain imaging results for contrast HC (thumb [pain-sensory] vs joint [pain-sensory]), and contrast HC vs FM (thumb [pain-sensory]) and HC vs RA (joint [pain-sensory]).

**Table 3:** Psychophysiological interaction functional connectivity analyses.

Psychophysiological interaction (PPI) Seeding from L IPL [MNI −62 −38 38]	x	y	z	k/E	*z*-score	FWE
FM > RA PPI from L IPL [pain-sensory]						
n/s						
RA > FM PPI from L IPL [pain-sensory]						
L precentral gyrus (M1), *encapsulating bilateral S1, L dlPFC, SMA*	−34	4	36	5466	4.63	<0.001
L parietal lobe/postcentral gyrus (S1)	−26	−46	54	430	3.98	0.016
L putamen *encapsulating anterior insula, IFG pars orbitalis*	−26	6	−8	332	3.95	0.039
FM PPI from L IPL [pain-sensory]						
n/s						
RA PPI from L IPL [pain-sensory]						
R postcentral gyrus (S1), *encapsulating L M1, R SMA, R S2, bilateral supramarginal gyrus*	38	−20	46	19489	5.14	<0.001
L inferior occipital lobe	−48	−70	−12	312	3.80	0.047

Seeding from R IPL [MNI 56 −24 24]	x	y	z	k/E	*z*-score	FWE
FM > RA PPI from R IPL [pain-sensory]						
n/s						
RA > FM PPI from R IPL [pain-sensory]						
R postcentral gyrus (S1)	54	−24	52	819	4.86	<0.001
R rolandic operculum (S2)	48	−18	20	1293	4.85	<0.001
FM PPI from R IPL [pain-sensory]						
n/s						
RA PPI from R IPL [pain-sensory]						
R rolandic operculum (S2), *encapsulating S1 and M1*	48	−18	18	2620	5.11	<0.001
L supramarginal gyrus, *encapsulating S2*	−58	−24	36	764	4.41	0.002

dlPFC, dorsolateral prefrontal cortex; FM, fibromyalgia; FWE, family-wise error; IFG, inferior frontal gyrus; IPL, inferior parietal lobe; MNI, Montreal Neurological Institute; PPI, psychophysiological interaction.

Pain-related cerebral brain activation in HCs in response to painful pressure at finger joint vs thumb (left hand), as well as group comparisons between HC vs FM (thumb [pain-sensory]) and HC vs RA (joint [pain-sensory]) can be found in supplementary table 1 (available at http://links.lww.com/PAIN/B421).

#### 4.2.1. Neuroimaging correlational results

**Figure 3.**
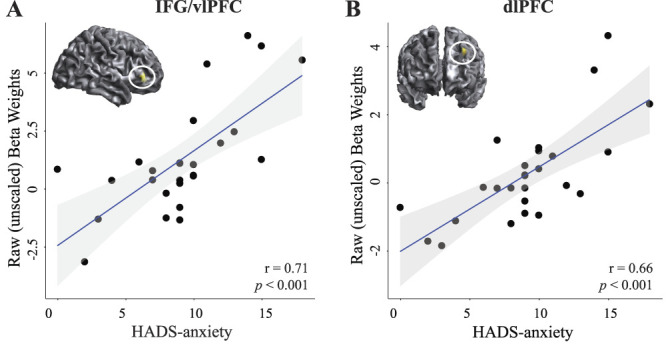
(A) Significant whole-brain activation during [Pain-Sensory] stimulation within the FM group that positively correlated with summarized anxiety scores of the hospital anxiety and depression scale (HADS-anxiety). Specifically, patients with FM with higher HADS-anxiety scores exhibited higher pain-related brain activation in (A) right IFG/vlPFC and (B) left dlPFC. Significant clusters showing a positive correlation between BOLD response and HADS-anxiety are displayed in yellow, marked with a white circle and displayed in the top left corner within the respective correlational plot. Correlational plots were plotted in *R* exclusively for visual purposes. The x-axis indicates HADS-anxiety scores and the y-axis indicates raw (unscaled) beta weights extracted from each significant cluster. Black dots within correlational plots indicate individual FM subjects. The depicted brain activation was derived from a whole-brain statistical map corrected for multiple comparisons using FWE correction *P*_*FWE*_ < 0.05 at an initial threshold of *P* < 0.001 uncorrected with 20 contiguously activated voxels. dlPFC, dorsolateral prefrontal cortex; FWE, family-wise error correction; IFG, inferior frontal gyrus; vlPFC, ventrolateral prefrontal cortex.

Correlational analyses can be found in Table 4. Specifically, patients with FM vs RA revealed a positive correlation between brain activation during [Pain-Sensory] and measures of anxiety (ie, FM > RA [pain-sensory] × HAD-anxiety) in right precuneus. Within patients with FM, increased measures of anxiety correlated with increased pain-related brain activation in the left dlPFC and right inferior frontal lobe (Fig. 3). No correlations were observed in patients with RA (Table 4). No significant correlations were found between brain activation during [pain-sensory] and disease duration (in months), HADS-d, FIQ (FM only), and HAQ (RA only). No significant contribution of sex or pressure intensity (P50 kPa) was observed on pain-related brain activation within or between groups. However, age was associated with significantly increased pain-related brain activation within the occipital lobe, thus included as a regressor of no interest throughout all brain imaging group analyses.

**Table 4:** Correlation between cerebral pain processing and anxiety scores.

Correlational analyses	x	y	z	k/E	*t*-value	*z*-score	FWE
FM > RA [pain-sensory] × HADanxiety							
R precuneus	12	−60	34	714	4.60	4.18	0.002
RA > FM [pain-sensory] × HADanxiety							
n/s							
FM [pain-sensory] × HADanxiety							
L dlPFC	−30	26	42	296	5.06	4.10	0.046
R IFG pars triangularis	48	32	2	357	4.94	4.04	0.025
RA [Pain-Sensory] × HADanxiety							
n/s							

dlPFC, dorsolateral prefrontal cortex; FM, fibromyalgia; FWE, family-wise error; IFG, inferior frontal gyrus.

## 5. Discussion

The aim of the current study was to directly compare cerebral pain modulation in well-characterized RA (without FM comorbidity) and FM (without RA comorbidity) patients. The results reveal distinct cerebral differences in response to evoked pressure pain between these 2 chronic pain cohorts. Specifically, patients with FM relative to patients with RA exhibited increased bilateral brain activation in the supramarginal gyrus (SMG) of the IPL; left IFG/ventrolateral prefrontal cortex (vlPFC) encapsulating left dlPFC; and right IFG/vlPFC when painfully stimulated at the left hand (thumbnail in FM, most inflamed finger joint in RA) (Fig. 1C and Table 2). However, patients with RA relative to patients with FM revealed increased task-based functional connectivity during painful pressure stimulation, between IPL and brain regions involved in the sensorimotor network (SMN; ie, M1, S1, S2) (Figs. 2A and B and Table 3), as well as between left IPL and the FPN (ie, dlPFC and parietal cortex) (Fig. 2A). Together, the current results suggest pain-related disruptions in patients with RA relative to patients with FM, in brain regions noted to be negatively affected by peripheral inflammation (ie, IPL and prefrontal cortex).^7,23,46,49,52^ However, patients with FM relative to patients with RA exhibited pain-related disruptions in functional connectivity that speculatively may reflect a discussed sensory disintegration in FM,^9,12,16,50,51^ or be explained by the higher inflammatory activity in the RA group. Finally, higher self-assessed anxiety scores (HADS-anxiety) were associated with increased dlPFC and vlPFC pain-related brain activation within patients with FM (Table 4) and highlights the complex interaction between sensory (brain activation during painful stimulation) and affective (anxiety scores) dimensions in this patient group.

### 5.1. The inferior parietal lobe in rheumatic conditions

The most prominent findings involved group differences in pain-related brain activation as well as altered functional connectivity of the right and left SMG of the IPL. Besides the role of SMG/IPL in language, this region also constitutes part of the somatosensory association cortex and is implicated in sensorimotor integration.^1,3,4^ Damage to this particular region can cause permanent alterations of pain and sensory sensations,^4^ result in disorders of proprioception and body awareness,^1,5^ and atrophy of the parietal lobes have been associated with interoceptive impairments.^16^

Accumulating data suggest a link between peripheral inflammation and detrimental effects on IPL structure and function. For example, a 3-year longitudinal study reported that higher levels of TNF-α was associated with reduced IPL brain volume.^7^ Cross-sectional studies have noted an association between TNF-α, IL-1β, and reduced brain volume,^52^ as well as between TNF-α and reduced FPN.^49^ In rheumatic conditions, altered resting-state functional connectivity of the IPL has been linked to elevated levels of inflammation (erythrocyte sedimentation rate) in patients with RA^23,46^ as well as in patients with RA with concomitant FM.^23^ Specifically, patients with RA with high levels of inflammation exhibited reduced IPL gray matter as well as increased connectivity between the left IPL and multiple brain networks such as default mode network, salience network, and FPN.^46^ Note that the latter is in alignment with the current study, ie, increased task-based connectivity between left IPL and FPN in patients with RA vs patients with FM. In patients with RA with concomitant FM, a positive relationship has been noted between higher levels of peripheral inflammation and increased functional connectivity between the left IPL to dACC, mPFC, right dlPFC, and right SMA; and between the insula and left IPL.^23^ The authors proposed that in some patients with RA, an interaction, and perhaps integration, of inflammation-linked brain connectivity and classic pronociceptive circuitry may lead to sensitization of CNS in a “top-down” manner.^23^ Based on this, we propose that the current observations of increased pain-related connectivity in RA vs FM between bilateral IPL and SMN and left IPL and FPN could be related to the inflammatory activity present in our RA, which is distinct from our patients with FM. Longitudinal studies could help elucidate whether these mechanisms may drive RA towards a nociplastic state. An alternative explanation is that the group difference reflects a sensory disintegration in FM.^9,16,50,51^ In support of the latter, we previously found reduced resting-state functional connectivity between the parietal lobe and somatosensory regions in the current cohort of patients with FM ^12^ as opposed to a pattern of sensory integration (ie, increased connectivity) in RA.^11^

### 5.2. The prefrontal cortex in rheumatic conditions

The current results of reduced dlPFC activation in RA vs FM during painful stimulation are in alignment with previous reports of aberrant pain-related dlPFC activation in patients with RA. Specifically, we previously reported diminished dlPFC activation in the current cohort of patients with RA during 2.5-second painful joint stimulation,^43^ whereas increased dlPFC activation has been observed during continuous (6 minutes) but less intense painful stimulation.^32^ Speculatively, the latter may reflect a stable ongoing inhibition via opioid signalling in response to continuous but lower painful stimulation intensity. However, when enough pain intensity is reached, the dlPFC may temporarily deactivate to protect the inflamed joints and to avoid additional harm. Alternatively, the reduced dlPFC activation may suggest that patients with RA attach less importance to painful stimuli from inflamed joints, which could reflect a form of habituation to commonly painful stimuli as every movement of inflamed joints is painful due to peripheral (and central) sensitization.

Conversely, the group difference could also be explained by an increased activation of the prefrontal cortex in patients with FM (ie, FM > RA [Pain-Sensory] exhibited more left dlPFC and bilateral IFG/vlPFC activation). The dlPFC is known for its involvement in cognitive aspects of pain perception such as increased attention towards painful stimulus and salience detection.^47^ Ventrolateral and dorsolateral prefrontal hyperactivation has been observed in FM subjects in response to pain stimuli onset and offset.^20^ Specifically, while the former correlated with unpleasantness ratings during pain onset, the latter correlated with catastrophizing scores during pain offset. Accumulating data suggest a link between maladaptive prefrontal processing and pain catastrophizing in patients with FM.^10,20,44^ In the current study, higher ratings of anxiety in patients with FM, but not in patients with RA, were associated with increased cerebral processing of pain within the frontal lobe (ie, left dlPFC and right vlPFC); however, the FM patient cohort also exhibited significantly higher ratings of anxiety compared to the RA cohort. Together, the combined results of increased dlPFC, vlPFC, and parietal activation may suggest that patients with FM compared to patients with RA exhibit more salience and attention to pain when exposed to painful pressure stimulation.

## 6. Limitations and strengths

In the current study, we compared brain activation in response to evoked pressure pain at 2 different locations on the left hand (ie, thumbnail and finger joint). This decision was based on previous studies using the same pressure pain apparatus that suggest the thumbnail may serve as a clinically relevant stimulation site in patients with FM^17,21^ but not in patients with RA,^43^ while no differences in cerebral processing after stimulation at these 2 sites was seen in HC.^43^ Patients with RA and HC were stimulated at 2 sites (ie, thumbnail and joint), whereas patients with FM were only stimulated at the thumbnail. Thus, although both groups received the same number of painful stimuli at the sites included in the analysis, the higher amount of pressure stimuli in patients with RA and HC could, hypothetically, increase central sensitisation. However, we consider this unlikely as the stimulation sites were alternated in a counterbalanced order between runs.

Furthermore, there was an unequal sex balance across the patient groups, but we did not find any significant effects of sex on pain-related brain activation. However, it should be noted that our results are not generalizable to male patients due to the low number of male subjects. Although there was a significant difference between the groups in ratings of anxiety and depression, all patients were below clinically relevant levels. Finally, we would like to highlight strengths with the current study. First, both RA and FM cohorts were well characterized and age-matched. Second, the data were collected during the same period, using the same pain applicator, experimental paradigm, facilities, and test leaders. Third, no significant differences were detected between the groups in clinically relevant characteristics such as age, body mass index, and clinical pain (VAS). Last, no confounding effects of sex, depression, or fatigue were observed.

## 7. Conclusion

The current results revealed distinct neurological patterns of altered cerebral pain processing in well-characterized FM and RA patients. Specifically, patients with RA vs patients with FM exhibited reduced pain-related brain activation in regions that have previously been noted to be particularly affected by peripheral inflammation (IPL and PFC). However, FM vs RA exhibited reduced pain-related functional connectivity between left IPL to FPN and SMN, as well as between right IPL and SMN. The group differences in functional connectivity could speculatively reflect inflammatory-related increases in connectivity in the patients with RA and/or a sensory disintegration (reduced/less stable connectivity) in FM. Furthermore, the results indicated that patients with nociplastic pain (FM) exhibit more pronounced pain-related activation in prefrontal areas (dlPFC and vlPFC) associated with salience and attention to pain, which in the current study could be explained by higher levels of anxiety in our patients with FM compared to patients with RA.

## Conflict of interest statement

The authors have no conflicts of interest to declare.

## Appendix A. Supplemental digital content

Supplemental digital content associated with this article can be found online at http://links.lww.com/PAIN/B421.

## Supplementary Material

SUPPLEMENTARY MATERIAL
